# The identification of six risk genes for ovarian cancer platinum response based on global network algorithm and verification analysis

**DOI:** 10.1111/jcmm.15567

**Published:** 2020-08-06

**Authors:** Linan Xing, Wanqi Mi, Yongjian Zhang, Songyu Tian, Yunyang Zhang, Rui Qi, Ge Lou, Chunlong Zhang

**Affiliations:** ^1^ Department of Gynecology Harbin Medical University Cancer Hospital Harbin China; ^2^ College of Bioinformatics Science and Technology Harbin Medical University Harbin China

**Keywords:** Fanconi anaemia, homologous recombination, ovarian cancer, platinum treatment

## Abstract

Ovarian cancer is the most lethal gynaecological cancer, and resistance of platinum‐based chemotherapy is the main reason for treatment failure. The aim of the present study was to identify candidate genes involved in ovarian cancer platinum response by analysing genes from homologous recombination and Fanconi anaemia pathways. Associations between these two functional genes were explored in the study, and we performed a random walk algorithm based on reconstructed gene‐gene network, including protein‐protein interaction and co‐expression relations. Following the random walk, all genes were ranked and GSEA analysis showed that the biological functions focused primarily on autophagy, histone modification and gluconeogenesis. Based on three types of seed nodes, the top two genes were utilized as examples. We selected a total of six candidate genes (FANCA, FANCG, POLD1, KDM1A, BLM and BRCA1) for subsequent verification. The validation results of the six candidate genes have significance in three independent ovarian cancer data sets with platinum‐resistant and platinum‐sensitive information. To explore the correlation between biomarkers and clinical prognostic factors, we performed differential analysis and multivariate clinical subgroup analysis for six candidate genes at both mRNA and protein levels. And each of the six candidate genes and their neighbouring genes with a mutation rate greater than 10% were also analysed by network construction and functional enrichment analysis. In the meanwhile, the survival analysis for platinum‐treated patients was performed in the current study. Finally, the RT‐qPCR assay was used to determine the performance of candidate genes in ovarian cancer platinum response. Taken together, this research demonstrated that comprehensive bioinformatics methods could help to understand the molecular mechanism of platinum response and provide new strategies for overcoming platinum resistance in ovarian cancer treatment.

## 1 INTRODUCTION

Ovarian cancer is the leading killer of gynaecological malignancy in women worldwide. In spite of debulking surgery and medical treatment of platinum, drug resistance is still the stumbling block to ovarian cancer therapeutics.^1^ Patients who have evidence of disease progression on primary therapy or after a treatment‐free interval of less than 6 months are considered platinum resistant, and those with evidence of relapse or develop progression after a treatment‐free interval of exceeding 6 months can be called platinum sensitive.^2^ After standard treatment, although more than 70% of patients respond to chemotherapy with cisplatin, a large number of patients will relapse and develop drug resistance within two years, with a survival rate of about 40%.^3^ Hence, exploring the molecular mechanisms underlying chemoresistance and identifying risk signatures are the key strategy to accelerate advancement in ovarian cancer therapy.^4^

Resistance to platinum‐based treatment can be intrinsic or acquired, and it is caused by a variety of mechanisms in ovarian cancer.^5^ The researchers have performed a large amount of low‐throughput experiments to analyse the resistance‐related mechanism and identify potential biomarkers for overcoming platinum resistance in ovarian cancer. For example, Wu et al discovered that Akt inhibitor SC66 was used in a NOD‐SCID xenograft mouse model and a group of eight ovarian cancer cell lines. They found that SC66 regulated collagen type XI alpha 1 chain by inhibiting Akt/mTOR signalling, and it could enhance cell sensitivity to drugs and inhibit proliferation/invasion.^6^ An additional study by Hu et al discovered that interleukin 17 receptor B (CRL4) was significantly increased in cisplatin‐resistant ovarian cancer cells, and knocking down CRL4 with shRNA reversed cisplatin resistance in ovarian cancer cells. CRL4 has been proved to play an important role in apoptosis and drug resistance by targeting baculoviral IAP repeat containing 3 (BIRC3) in ovarian cancer cells.^7^ However, these studies were performed at the low‐throughput level for identifying single gene signature. Moreover, it remains unclear whether the mechanism is universal in all patients, because of the small number of tissue samples in researches.^8^, ^9^ Therefore, it is necessary to employ a large number of tissue samples and integrate multiple sets of data for analysis in the research.

Homologous recombination (HR) and Fanconi anaemia (FA) are two of the major DNA repair pathways, and some researches have indicated that both of these pathways are related to platinum resistance in ovarian cancer. It is well known that the main target of platinum agents is DNA, which mainly plays a role in DNA damage, thereby activating the DNA damage response.^10^ However, if DNA fails to repair the damage, tumours or activated cell death will occur. Moreover, changes in these repair pathways will contribute to the tumour sensitive or resistant to platinum agents.^11^ HR is an error‐free DNA repair system that is activated in the case of DNA double‐stranded damage.^12^ In the past few years, more than 50% of patients with high‐grade serous ovarian cancer have been proven to have defects in HR repair. Because of the existence of this defect, this tumour type has a very high sensitivity to platinum agents.^13^ Studies have shown that patients with HR deficiency have clinical manifestations of visceral recurrence, slightly younger age at diagnosis and better sensitivity to platinum agents.^14^ As for FA, it is a hereditary disease that can cause cancer susceptibility. FA pathway facilitates the monoubiquitination of the complementation of FA complementary group D2‐FA complementary group I heterodimer, thereby activating DNA damage response. Previous studies have revealed that the mutations in the FA gene result in the cell being highly sensitive to platinum agents.^15^ A recent research demonstrated that acquired disruption of FA pathway bought about chromosomal instability and hypersensitivity to cisplatin in ovarian tumours. Destruction of this pathway could create cisplatin sensitivity, and recovery of it would generate cisplatin resistance.^16^ However, the inner associations between HR and FA pathways, and key risk genes within these functions were not explored.

Recently, many researches were performed based on global network to explore the complex biological mechanism involved in ovarian cancer. Through public databases, Wang et al obtained the data on lncRNAs, mRNAs and miRNAs with differential expression, compared with normal ovarian tissue and epithelial ovarian cancer. They used the bioinformatics method to predict interactions of lncRNAs, mRNAs and miRNAs and then built the LINC00284‐related ceRNA network. Based on biological function analysis, they found that the LINC00284‐related ceRNA network was related to epithelial ovarian cancer carcinogenesis, and finally confirmed that LINC00284 was a new potential prognostic biomarker for epithelial ovarian cancer.^17^ In another study, authors downloaded three sets of expression profiles from the Gene Expression Omnibus (GEO) database, containing information on ovarian cancer tissues and normal tissues. A total of 190 differentially expressed genes were identified. The protein‐protein interaction (PPI) network was constructed by the identified differentially expressed genes. Ultimately, the study identified the 17 most closely related genes among differentially expressed genes from the PPI network.^18^ Network‐based random walk algorithm was developed to identify candidate genes by use of a global network distance measure.^19^ This algorithm not only provides an improved method for risk gene selection but also added core seed genes integration framework in global mechanism exploration.

In this study, we first acquired the HR‐related pathway genes and FA‐related FANC‐BRCA pathway genes in the Molecular Signatures Database (MsigDB) and classified these genes into three types, including HR only gene(HO‐G), HR/FA common gene (HFC‐G) and FA only gene (FO‐G). Secondly, we randomly walked three types of genes and seed nodes in the complex disease‐specific gene‐gene network to optimize risk genes. According to the random walk results, the top two genes from each seed node were selected as instances of candidate genes' verification analysis. Finally, a total of six candidate genes were analysed and verified to different degrees in multiple databases. Notably, the quantitative real‐time polymerase chain reaction (RT‐qPCR) assay was performed to verify the differential expression of mRNA levels in cisplatin‐resistant and cisplatin‐sensitive ovarian cancer cell lines.

## 2 MATERIALS AND METHODS

### 2.1 Publicly expression data sets and signature genes

The expression data sets were downloaded from the public database, TCGA database (https://portal.gdc.cancer.gov/) and GEO database (https://www.ncbi.nlm.nih.gov/gds/). The TCGA database covers the molecular characteristics of more than 20,000 primary cancers. It analyses and provides genome sequence, expression, methylation and copy number variation data of more than 11 000 individuals in redundant 30 different types of cancer.^20^ The expression matrix with ovarian tumour type was directly obtained TCGA database to construct the ovarian cancer‐specific co‐expression network.

The GEO database archives microarrays and other forms of high‐throughput functional genomics data. We searched for the keyword ‘ovarian cancer’ in it to obtain relevant studies with platinum‐resistant and platinum‐sensitive information. The inclusion criteria included: (a) The number of samples was not less than 8, and after grouping, each group of samples was no <4; (b) the sample was treated with platinum and did not undergo neoadjuvant chemotherapy; and (c) the classification of the sample was reliable. (The classification method had literature support.)

And finally, the three GEO data sets were screened out as validation sets. The access number of three GEO data sets was GSE45553, GSE51373 and GSE15622. The normalized expression data in a series matrix format and relevant clinicopathological information were retrieved from GEO. GSE45553 had 8 samples, including 4 platinum‐resistant samples and 4 platinum‐sensitive samples. GSE51373 had 28 samples, including 12 platinum‐resistant samples and 16 platinum‐sensitive samples. GSE15622 contained 69 samples, of which 33 patients used carboplatin, 18 sensitive samples and 15 resistant samples. The probe set without specific gene annotation was filtered for each sample. For all the expression data, the average expression value was calculated as the final value for duplicated samples. The HR genes and FA‐related genes were obtained from the C2 class of MsigDB v7.0 (https://www.gsea‐msigdb.org/gsea/msigdb/genesets.jsp).

### 2.2 The integrated complex network

We integrated the PPI network and ovarian cancer co‐expression network to form the complex network. The data of PPI networks^21^ were obtained from previously contained 12 databases, including BioGRID, DFCI_NET_2016, HI‐II network, HPRD, InnateDB, INstruct, IntAct, KinomeNetworkX, MINT, PhosphositePlus, PINA and SignaLink2.0. In addition, we constructed the integrated networks with the interaction from at least two databases. This PPI network contained 15 913 gene nodes and 256 197 interaction edges. We further constructed the co‐expression network based on the TCGA ovarian cancer data set. For each gene pair, we calculated the Spearman correlation value and set the absolute 0.6 as the cut‐off. Then, the ovarian cancer‐specific co‐expression network was formed which contained 11 105 gene nodes and 173 488 co‐expression edges. The final integrated network contained 15 913 nodes and 429 685 edges.

### 2.3 Random walk algorithm based on integrated network

Based on the global combined network which contained both PPI interaction and co‐expression relations, we further performed a global risk impact analysis to optimize mRNAs by using the random walk algorithm. The random walk algorithm was developed and utilized for multiple types of disease mechanism analysis and displayed more advantages in risk or prognostic genes identification^19^, ^22^, ^23^ on the basis of the global network. Based on the reconstructed network mentioned above, the functional genes from HR/FA pathways were regarded as seed nodes. Considering the difference between these two functions, we, respectively, annotated each of the three types of genes (HO‐G, HFC‐G and FO‐G) into this global network and the corresponding annotated genes were treated as seed nodes. The random walk algorithm was then used to evaluate the global risk impact of seed nodes on each component as follows:

$$P^{t+1}\;=\;\left(1\;‐\;r\right)WP^{t}\;+\;rP^{0},$$

where *W* is the column‐normalized adjacency matrix of the global integrated network, which consisted of 0 and 1. *P^t^* was a vector, in which a node in the global network held the probability of finding itself in this process up to step *t*. The initial probability vector *P* ^0^ was constructed in such a way, where equal probabilities were assigned to all seed nodes and the sum of their probabilities was equal to 1. Additionally, the restart of the walker at each step was the probability *r* (*r* = .7). When the difference between *P^t^* and *P^t^* ^+1^ fell below 10^−6^, the probabilities reached a steady state. Finally, each gene in the global network was given a score according to the values in the steady‐state probability vector *P^∞^*. In this study, the random walk process was performed three times to, respectively, obtain different optimization order for all genes.

### 2.4 Enrichment analysis

Gene ontology (GO) and Kyoto Encyclopedia of Genes and Genomes (KEGG) enrichment analysis was performed with GSEA function in clusterProfiler R package.^24^ The ordered gene list after random walk algorithm was treated as an input file, which reflected the comprehensive impact of seed nodes based on network topology. And false‐discovery rate adjusted *P* values were calculated by using Benjamini‐Hochberg correction.

### 2.5 Oncomine analysis

Oncomine (www.oncomine.org) is a database with powerful functions for analysing expression differences, which includes 715 gene expression data sets from 86 733 cancer tissues and normal tissues.^25^ In this study, TCGA, Yoshihara, Adib, Bonome, Welsh, Hendrix and Lu Ovarian^26^, ^27^, ^28^, ^29^, ^30^, ^31^, ^32^ were used to analyse the differential expression of the six candidate genes. The raw data downloaded from the Oncomine database were plotted as box plots using GraphPad Prism 7, and the *P* < .05 was considered to be significantly different.

### 2.6 UALCAN analysis

UALCAN (http://ualcan.path.uab.edu/) is a database for deep mining of TCGA and Clinical Proteomic Technology Assessment for Cancer data, which can be utilized to analyse gene or protein expression levels and correlations.^33^ UALCAN allows querying the expression pattern of the gene between tumour and normal tissues. It can also analyse the relative expression in different cancer stages, different populations and tumour subgroups and other clinicopathological features. In this study, we employed the analysis function of the UALCAN database to compare the grades and other clinical features for the six candidate genes at both mRNA and protein levels.

### 2.7 cBio Cancer Genomics Portal analysis

The cBio Cancer Genomics Portal (c‐BioPortal) (http://cbioportal.org) is an open‐access resource for interactive exploration of multiple cancer genomics data sets.^34^ In the TCGA PanCancer Atlas study, we searched the parameters of mutations, CNV and mRNA expression and then performed an in‐depth analysis of the six candidate genes. The Network tab visualized the interaction network of the six candidate genes and their neighbouring genes. We also selected the neighbouring genes with a mutation frequency greater than 10% for enrichment analysis using DAVID webtool (https://david.ncifcrf.gov/home.jsp). And the GO biological process (BP) and KEGG were considered in enrichment analysis. Cytoscape (version 3.5.1) was utilized to build and visualize the gene‐function networks of enrichment analysis results by DAVID.

### 2.8 KM‐Plotter database

Kaplan‐Meier Plotter (KM‐Plotter) database (http://kmplot.com/analysis/) is widely used to analyse the clinical effects of individual genes on the survival rate of different cancer types, mainly for the survival discovery and validation of meta‐analytic markers.^35^ We explored the research on ‘ovarian cancer’ in the database. In this research, we analysed the clinical effects of both overall survival and disease‐free survival for six candidate genes. It is worth noting that we compared the survival analysis of all patients in the study with those who had undergone platinum‐based chemotherapy. In all survival analysis, we considered *P* < .05 as a significant result.

### 2.9 Cancer cell lines and culture

Human ovarian cancer cell line A2780 and cisplatin‐resistant cell line A2780DDP were purchased from Shanghai Chuanqiu Biotechnology Co., Ltd. The A2780 and A2780DDP cell lines were cultured in RPMI (Beijing Labgic Technology Co, Ltd) 1640 with 10% foetal bovine serum in a humid incubator containing 5% CO_2_ at 37°C.

### 2.10 RT‐qPCR

The mRNA expression of candidate genes of the human ovarian cancer cell line (A2780 and A2780DDP) was determined by RT‐qPCR. The total RNAs were isolated by nucleic acid purification kit (AxyPrepTM Multisource Total RNA Miniprep Kit; Corning Life Science Co., Ltd) according to the manufacturer's instructions. One milligram of RNA was synthesized to cDNA using a Transcriptor First Strand cDNA Synthesis Kit (Cat. No. 04 879 030 001; RocheMolecular Systems, Inc). To quantify mRNA expression, qPCR was performed with the StepOnePLusTM Real‐Time PCR System (Applied Biosystems) and SYBR‐Green assay kit (Roche Diagnostics Gmbh) following the manufacturers' instructions. GAPDH was used as an internal control. Each of the 40 PCR cycles consisted of 10 min of pre‐denaturation as well as 15s of denaturation at 95°C and 1 min of annealing and extension at 60°C. The primer sequences are provided in Table S1. We calculated the level of expression of six candidate genes in the cells by the 2^−ΔΔCT^ method.

## 3 RESULTS

### 3.1 Optimization of candidate genes based on HR/FA function and integrated network

**FIGURE 1:**
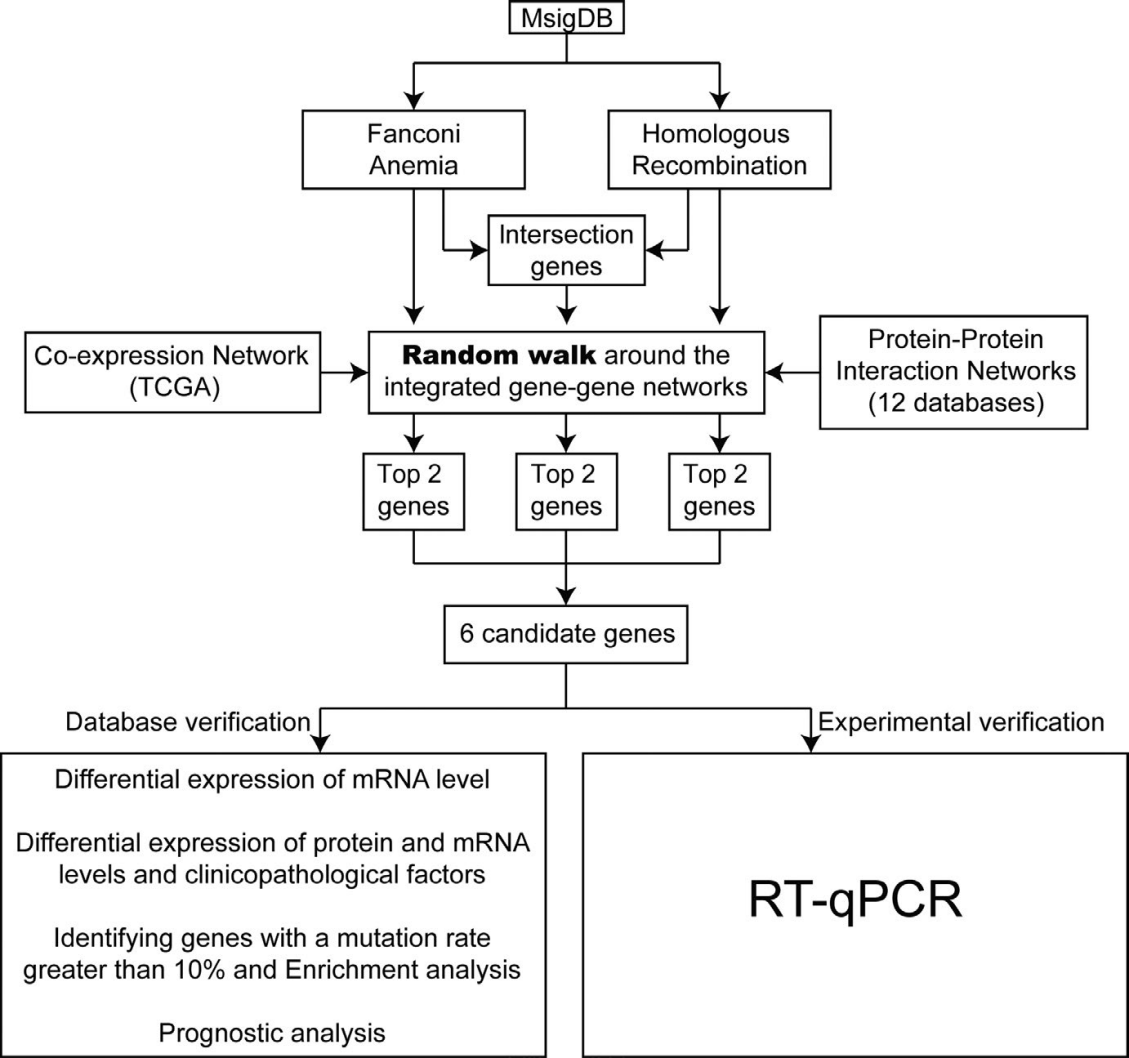
The overall workflow

To investigate platinum resistance in ovarian cancer, 56 genes of the FA‐associated genes and 53 genes of HR‐associated genes were extracted and intersected. As a result, 15 of these genes shared by FA and HR were regarded as HR genes (HFC‐G), indicating the close relationship between these two functions. Forty‐one genes were only associated with FA‐associated genes, but not with HR (FO‐G), and 38 genes were reversed (HO‐G). Furthermore, we integrated the PPI network from 12 databases and gene co‐expression network calculated based on TCGA data set (see Section 2). The robustness of PPI interactions was investigated using integrated networks with the interaction from at least two databases. Finally, these three types of genes were, respectively, regarded as seed genes and annotated into the integrated network. The random walk algorithm was performed three times to select the candidate genes (see Section 2). And detailed random walk results are provided in Table S2. The overall workflow of this study is shown in Figure 1.

### 3.2 GSEA functional analysis of candidate genes

**FIGURE 2:**
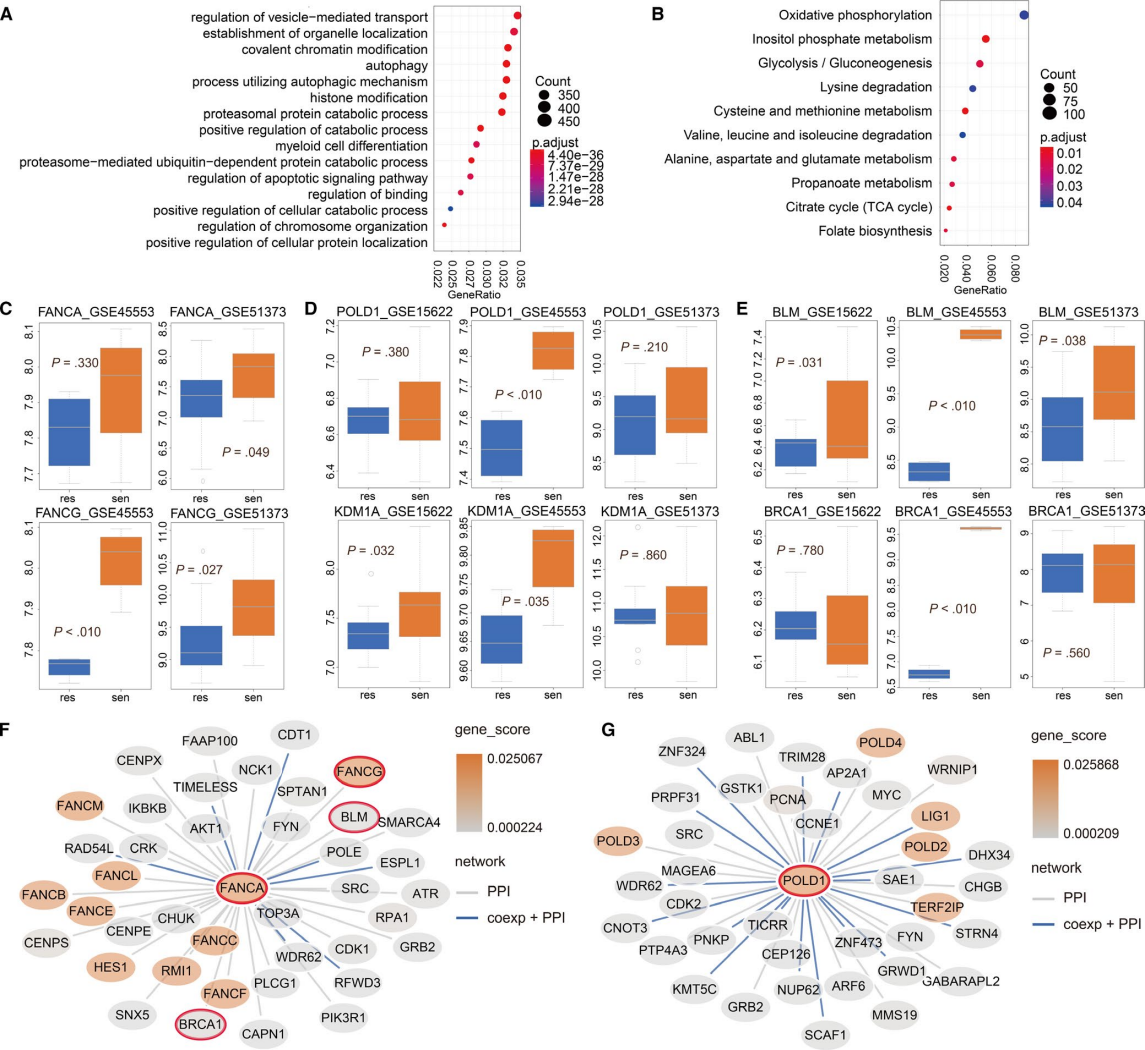
Functional analysis and verification of six candidate genes. (A) Enrichment analysis results of BP, (B) enrichment analysis results of FO‐G KEGG pathway. (C) FANCA and FANCG in the verification set GSE45553 and GSE51373, (D‐E) POLD1, KDM1A, BLM and BRCA1 in the verification set GSE15622, GSE51373 and GSE45553, and PPI direct interaction network of FANCA (F) and POLD1 (G)

Each gene involved in the network scored from 0 to 1 after the random walk, suggesting the association with seed functions. To further understand the biological functions driven by seed nodes, all genes were ranked in descending order and then the genes with a score of 0 were eliminated. Functional analysis of BP and KEGG to the ordered gene using GSEA analysis was subsequently performed. As shown in Figure 2A, the result revealed that three types of ordered genes were all enriched in many important BP terms, including autophagy, autophagy mechanism, histone modification and active regulation of catabolic processes. These biological functions are closely related to the examination of platinum resistance in ovarian cancer.^36^ As shown in Figure 2B, the result of KEGG, there were many significant biological pathways from FO‐G, such as inositol phosphate metabolism, glycolysis/gluconeogenesis, cysteine and methionine metabolism. Some researchers have studied the platinum resistance of ovarian cancer from the perspective of glucose metabolism and found that the glucose metabolism pathway has an important effect on overcoming platinum resistance of ovarian cancer.^37^ The similar KEGG results were also observed for HFC‐G and HO‐G (see Figure S1).

### 3.3 Independent validation of candidate genes in GEO database

Three independent GEO validation sets with available information of platinum response (see Section 2) were obtained to test the performance of the candidate genes from three types of seed nodes. We calculated the –log(*P*) mean of the three types of candidate genes in each data set, respectively. Based on the average of the three sets of data sets, we comprehensively obtained the average again. It can be seen from Figure S3 that the top ten genes all displayed good effects. The results of the genes ranked 1, 2, 4, 8 were significant, and the scores of the 5th and 6th genes were close to meaningful. As the good results were provided with the top two genes, we took the top two genes from three types of seed node as examples for the subsequent verification of candidate genes.

FA complement group A (FANCA), FA complement group G (FANCG), DNA polymerase delta 1 (POLD1), lysine demethylase 1A (KDM1A) human RecQ helicase (BLM) and breast cancer susceptibility gene 1 (BRCA1) were the these six candidate genes, and the detailed results are shown in Figure 2C‐E. It was recognized that compared with the resistant group, the expression level of the six candidate genes was increased in the sensitive group, indicating that these candidate genes were platinum‐sensitive genes. Additionally, we plotted the direct interaction network for two genes, FANCA and POLD1, as an example. As shown in Figure 2F,G, the most interaction genes of FANCA and POLD1 were also the seed genes. Therefore, these seed genes produced more impacts on each other from both co‐expression relations and PPI interactions.

### 3.4 mRNA‐ and protein‐level differential expression analysis of candidate genes

**FIGURE 3:**
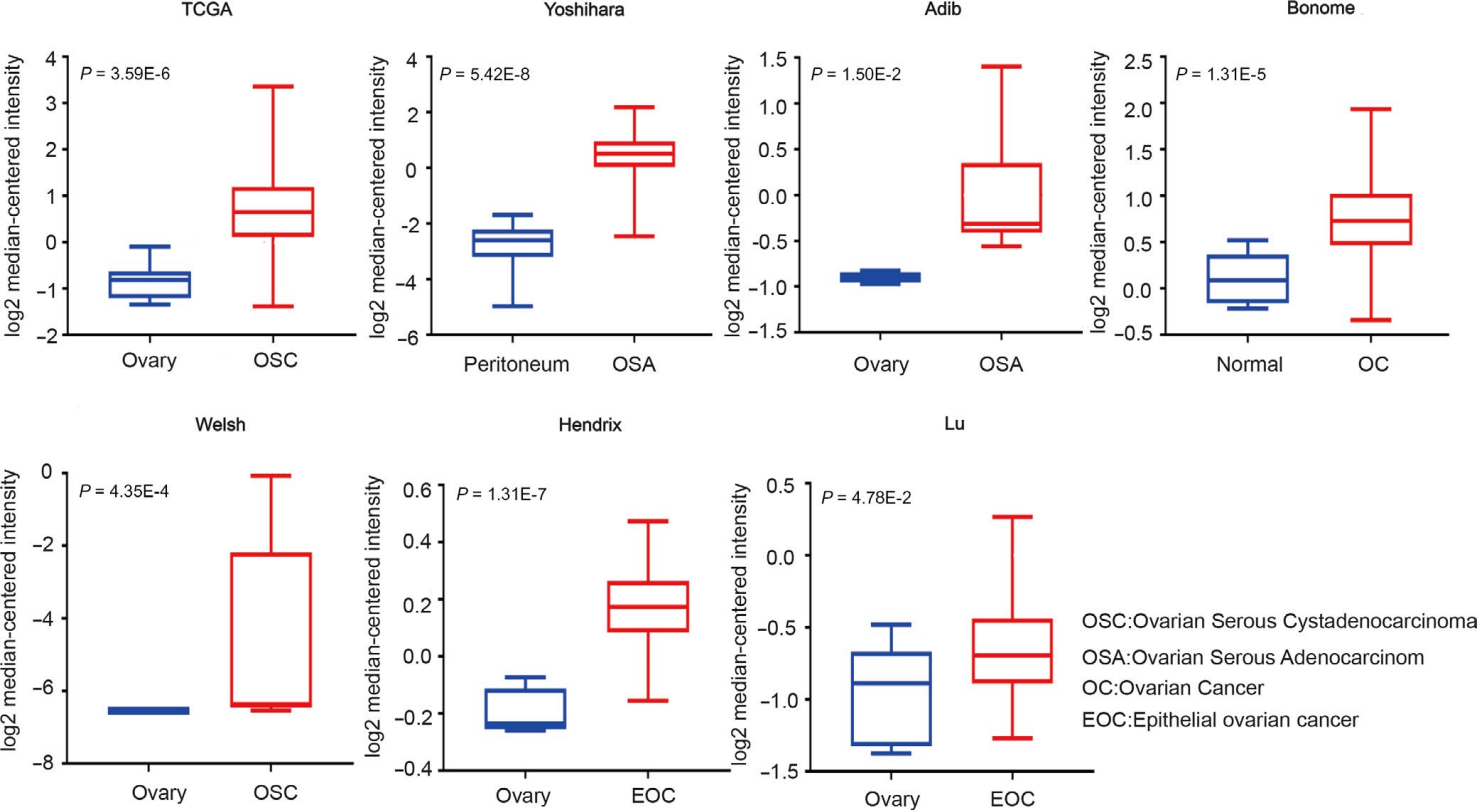
BLM transcription in Oncomine database

The differential expression analysis for the six candidate genes was employed by the Oncomine and UALCAN databases. A total of seven available studies were in the Oncomine database, and distinct studies have been conducted for different these genes. All the results are shown in Table 1. In detail, BLM was overexpressed in ovarian cancer tissues in the seven studies (see Figure 3). BRCA1 showed significant differences between ovarian cancer tissues and normal tissues in TCGA, Bonome and Yoshihara. FANCA was overexpressed in six databases other than LU, and POLD1 was highly expressed in in cancer tissues in six databases other than HENDRIX. According to Table 1, the expression of FANCG was significantly different in TCGA, Bonome, Yoshihara and Adib Ovarian. KDM1A up‐regulated in ovarian cancer tissues of TCGA and Bonome, and the difference in Bonome was the most obvious, with the *P* value of 2.14E‐8.

**TABLE 1:** Six candidate genes' transcription in Oncomine database

	TCGA	Bonome	Yoshihara	Adib	Welsh	LU	Hendrix
BLM	3.95E‐6	1.31E‐5	5.42E‐8	1.50E‐2	4.35E‐4	4.78E‐2	1.31E‐7
BRCA1	6.00E‐3	2.00E‐3	8.21E‐5	‐	‐	‐	‐
FANCA	7.55E‐5	2.28E‐7	2.76E‐10	1.50E‐2	1.01E‐6	‐	1.42E‐2
FANCG	1.43E‐4	1.00E‐3	3.10E‐5	2.50E‐2	‐	‐	‐
KDM1A	8.51E‐5	2.14E‐8	‐	‐	‐	‐	‐
POLD1	4.92E‐7	1.98E‐4	3.74E‐10	4.0E‐2	1.01E‐6	1.19E‐2	‐

**FIGURE 4:**
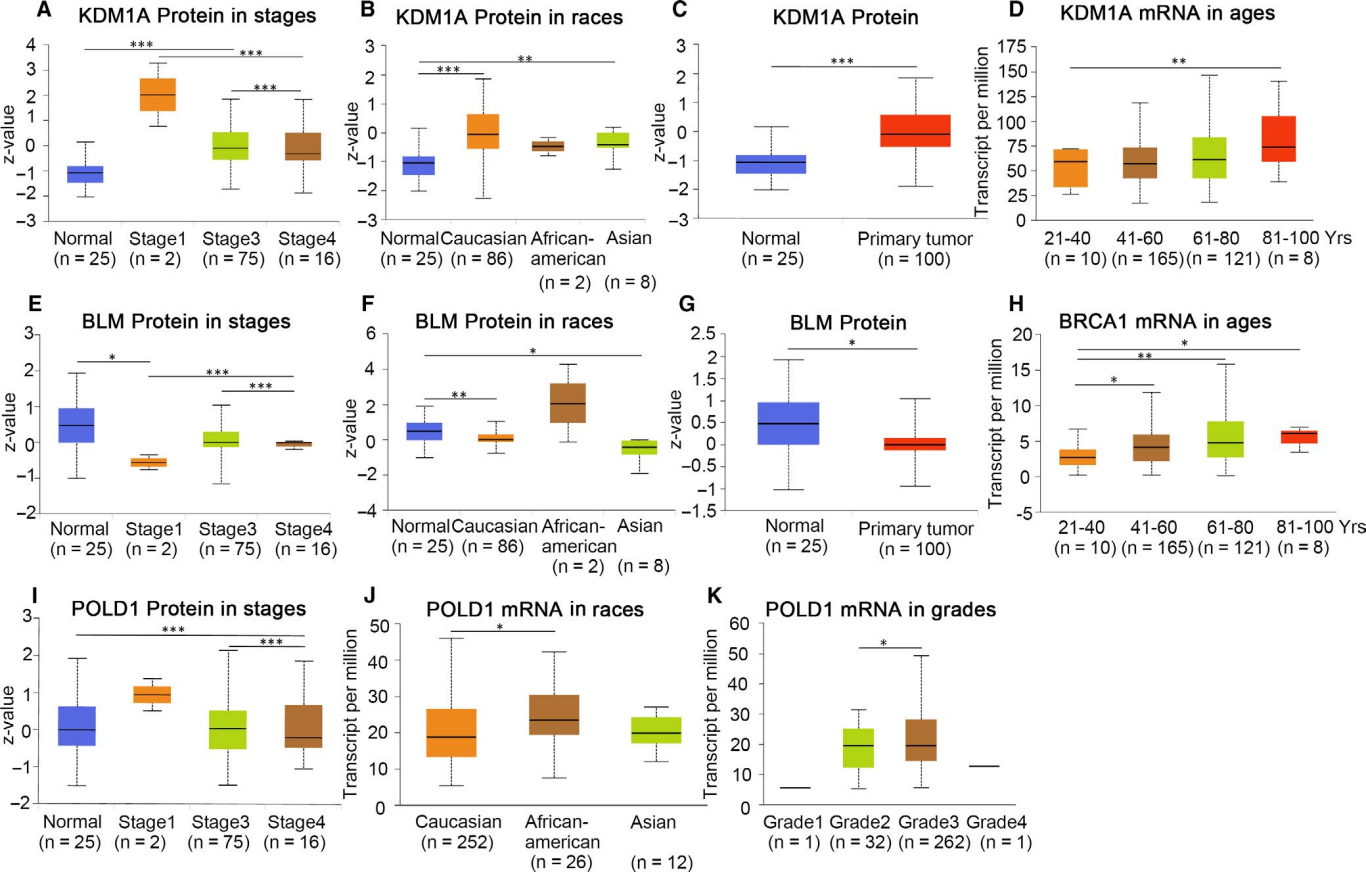
Four candidate genes' protein and transcription in multivariate clinicopathological subgroup analysis using UALCAN. (A‐D) protein and mRNA expression of KDM1A in normal and ovarian cancer samples with different stages, races and ages. (E‐G) protein expression of BLM in normal and ovarian cancer samples with different stages and races. (H) mRNA expression of BRCA1 in normal and ovarian cancer samples of different ages. (I‐K) protein and mRNA expression of POLD1 in normal and ovarian cancer samples with different stages, races and grades. *P < .05; **P < .01; ***P < .001

Furthermore, by using the UALCAN database, we performed a multivariate clinical pathology subgroup analysis, and the detailed results of four candidate genes are demonstrated in Figure 4. Taking KDM1A as an example, the protein expression level of KDM1A in ovarian cancer tissues was significantly higher than that in normal tissues (Figure 4C). As shown in Figure 4A, there were significant differences between the three groups in the stage. It could not be confused from Figure 4B that the two groups were significantly different in the ethnic grouping. And compared with the 80‐100‐year‐old group, KDM1A mRNA expression was decreased at the age of 21‐40 years old (Figure 4D). In conclusion, the differential expression analysis from both mRNA and protein levels indicated that these candidate genes are important biomarkers for predicting unfavourable biological behaviour in ovarian cancer formation and development.

### 3.5 Mutation‐driven network and survival analysis of candidate genes

**FIGURE 5:**
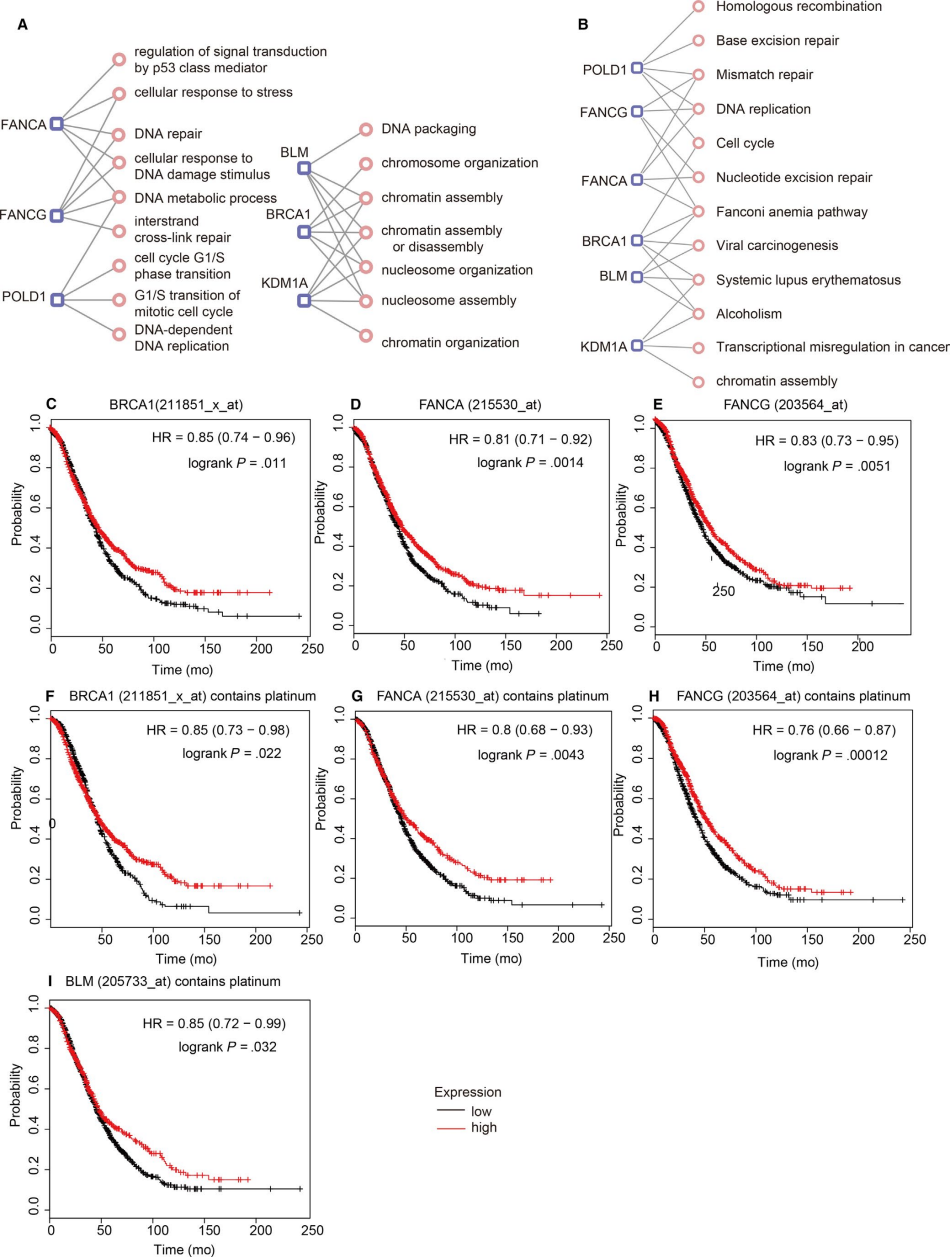
Enrichment analysis of neighbouring genes with mutations greater than 10% and prognosis analysis. The gene‐function network (A) BP and (B) KEGG. (C‐M) KM‐Plotter of candidate genes in all ovarian cancer patients and platinum‐treated patients

Next, we made a thorough inquiry to the bio‐interaction network and survival analysis of the six candidate genes. In the current study, the ‘network’ function in the c‐BioPortal database was used to screen out neighbouring genes which mutations exceeded 10% of the six candidate genes, whereas BP and KEGG enrichment analyses were also assessed for these genes by DAVID software (Tables S3 and S4). The results showed that three of the candidate genes (BLM, BRCA1 and KDM1A) were mainly located in nucleosome tissues, and they were mainly involved in chromatin assembly, chromatin assembly or disassembly and nucleosome assembly. FANCA, FANCG and POLD1 were mainly involved in DNA metabolism processes (see Figure 5A). In the KEGG pathway analysis, there were more than three genes enriched in these pathways in mismatch repair, DNA replication, Fanconi anaemia pathway, systemic lupus erythematosus and alcoholism (see Figure 5B).

To assess the association between candidate genes and the prognosis of ovarian cancer patients, we utilized the KM‐Plotter database to analyse all ovarian cancer patients and patients with platinum. As shown in Figure 5, we observed that patients with overexpression BRCA1 and FANCA had better overall survival. After platinum treatment, their significance *P* value was higher than the overall patient with ovarian cancer in the database. Patients with high FANCG expression had significantly higher overall survival than patients with low expression. Patients who overexpressed FANCG treated with platinum had lower overall survival than all patients in the database, and the *P* value was .00012. Overexpression of BLM after platinum treatment had higher overall survival than patients with low expression with the *P* value of .032. By comparison, these four candidate genes were found to have better overall survival in patients with overexpression. Regarding disease‐free survival, we can discover from Figure S2 that patients treated with platinum expressing FANCA, FANCG and BLM had more significant specific *P* values than overall patients with ovarian cancer.

### 3.6 Low‐throughput RT‐qPCR analysis of candidate genes

**FIGURE 6:**
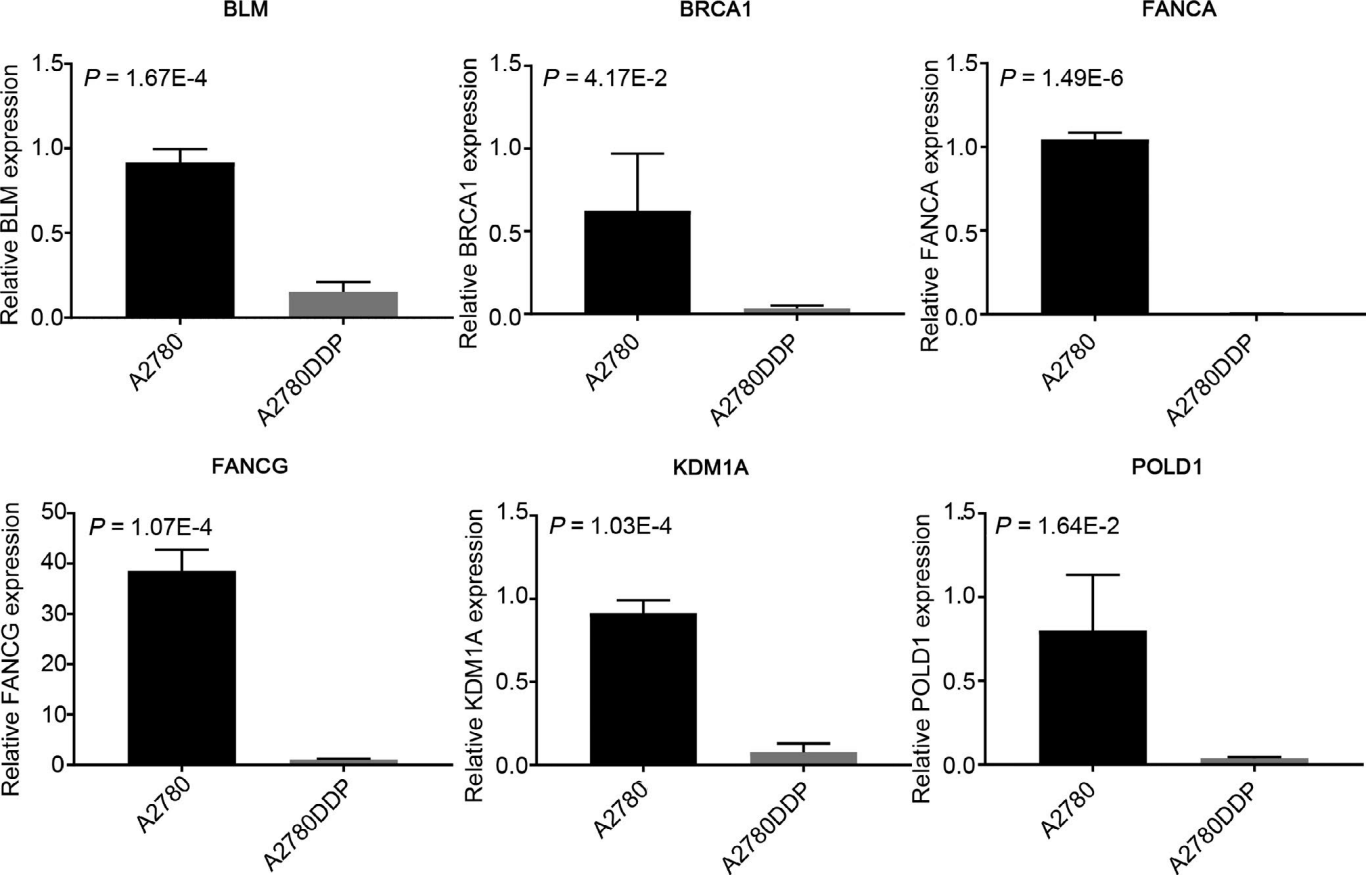
RT‐qPCR analysis of six candidate genes in the chemosensitive ovarian cancer cell lines (A2780) and A2780 cisplatin‐resistant cell lines (A2780DDP)

Ultimately, to further verify the expression difference of the six candidate genes in ovarian cancer platinum response, we tested the mRNA levels of six candidate genes in ovarian cancer cell lines, A2780 and A2780DDP by RT‐qPCR (see Section 2). As shown in Figure 6, the six candidate genes all displayed meaningful results in RT‐qPCR assay. For example, compared with two cell lines, BLM was overexpressing in A2780, and the *P* value was 1.67E‐4. BRCA1 expression was lower in A280DDP than A2780, with the *P* value of 4.17E‐2. FANCA's result was the most meaningful, and the *P* value was 1.49E‐6. KDM1A expression in A2780DDP was down‐regulated compared with A2780, and the *P* value was 1.03E‐4. The *P* value of FANCG was 1.07E‐4. As for POLD1, decreased expression was significantly in A2780DDP, with the *P* value of 1.64E‐2. The results of the low‐throughput experiment confirmed that these six candidate genes were all highly expressed in platinum‐sensitive cell lines, which is highly consistent with our previous results from GEO validation. This suggests that candidate genes are expected to become new biomarkers for overcoming platinum‐resistant issues for ovarian cancer.

## 4 DISCUSSION

Platinum is the main chemotherapy for advanced ovarian cancer; however, the drug resistance still deeply afflicts most patients and clinicians. Therefore, it is urgent to overcome the platinum‐based chemotherapy resistance of ovarian cancer and identify platinum response‐related biomarkers. In this study, considering that HR and the FA‐related FANC‐BRCA pathway have been previously extensively quested to be associated with ovarian cancer platinum response mechanisms, we utilized these two pathways as the origin. These genes were further classified into three groups for subsequent network optimization. To date, we provided high‐throughput network optimization algorithms to analyse platinum‐response studies in ovarian cancer.

We performed the random walk algorithm based on these three types of genes as seed genes, integrated network (including PPI and co‐expression relationships) and established three scoring matrices for candidate gene selections. The GSEA functional analysis was employed for enrichment analysis of three types of genes. And it is gratifying that the GSEA enrichment results (including BP and KEGG) for three types of random walk results were consistent, which were also related to platinum response in ovarian cancer.

Through the scoring matrix, we screened out the three types of seed nodes as candidate genes and detected the differential expression in three data sets with ovarian cancer platinum response information from GEO database. The top ten genes revealed reliable results to varying degrees. From Figure S3, it was not difficult to observe that the first and second genes displayed a rising polyline, whereas the third gene had a lower biological significance than the top two, showing a declining polyline. Therefore, we served the top two genes in the three scoring matrices as instances. And the six candidate genes were selected for verification and analysis. The results obtained in the current study demonstrated that expression levels of the six candidate genes were different in the most validation set, unfortunately, because FANCA and FANCG had no expression values in GSE15622 and not verified in this data set.

Many researchers have studied in six candidate genes to varying degrees in ovarian cancer. BRCA1 is one of the most common ovarian cancer genes in the process of HR repair of double‐stranded DNA breaks.^38^ Increasing numbers of studies have demonstrated that BRCA1 mutations increase the risk of ovarian cancer.^39^ The study has pointed out that serous ovarian cancer was sensitive to platinum because of a functional defect caused by insufficient BRCA1 levels. Patients lacking BRCA1 had a better chemotherapy response; however, reactivation of BRCA1 mutations might be the basis of platinum resistance in end‐stage patients.^40^ Genetic and functional evidence has suggested that BRCA1 is the major determinant of platinum response in HR DNA repair systems, and functional HR systems and intact BRCA1 functions are usually associated with platinum chemotherapeutic agents and PARPi enzyme inhibitors that in HGSOC cells.^41^ Giovanna and Massimo stated that a back mutation in the BRCA1 mutant gene or a deletion in the gene could restore the protein reading frame, thereby producing a functional protein and regaining HR levels, thus making the cell resistant to cisplatin and PARP inhibitors.^11^ In addition, Ganzinelli et al performed a univariate analysis in 171 cases of ovarian tumours, which showed that the increased expression of BRCA1 was associated with progression‐free survival. In patients with BRCA1 overexpression, it can be observed that there is an improvement in progression‐free survival in patients treated with platinum compared with patients treated with platinum and paclitaxel. However, patients with higher BRCA1 levels who were treated with platinum and paclitaxel had a longer overall survival compared with patients who were only given platinum.^42^

There is increasing evidence that the resistance to cross‐linking agents (such as cisplatin) is related to the functional status of the FA/BRCA pathway in cancer cells. The different absence of FA core components makes the cells have distinct sensitivities to cross‐linking agents.^43^ FANCA is the most mutated gene in FA cases, and FANCA acts as a multifunctional protein in the physiological role of FA/BRCA1 pathway repair interchain cross‐linking and HR.^44^ The researchers found that carriers of FANCA mutations were significantly associated with breast and ovarian cancer risks.^45^ In the study, the expression of 11 genes involved in the DNA repair pathway, including FANCA, was detected by RT‐qPCR. The results showed that FANCA expression was higher in stage I ovarian cancer than in stage III. Almost all DNA repair genes have low expression in stage III, indicating that as the disease progressed, while maintaining the metastatic potential, the different regulation of these genes leads to reduced expression.^42^ In the research of Kramer et al, it was shown that by jointly inhibiting DNA repair and cell cycle control mechanisms, HSP90 inhibitor ganetespib blocked the degradation of FANCA and the imbalance of nuclease DNA2. On this basis, ganetespib enhanced the anti‐tumour efficacy of carboplatin, thereby triggering overall chromosomal destruction, abnormal mitosis and cell death in ovarian cancer cells.^46^ It has been reported that heterozygous carriers of FA gene mutations will not cause FA phenotype, but may increase the risk of cancer. In addition to mutations, the epigenetic silencing of wild‐type FA gene expression appears to be important in certain cancer types. The report pointed out that FANCG and FANCA heterozygous mutations were susceptible to haematological malignancies and pancreatic cancer. And the lack of FANCA and FANCG expression was also associated with these two sporadic cancers.^47^ The FANCG gene mutation is the third most common type of FA mutation.^48^ FANCG and HR also have a certain functional relationship, and FANCG inactivation will reduce HR repair function.^49^

BLM is one of the genes responsible for Bloom syndrome and belongs to the RecQ family of DNA helicases.^50^ The enzyme is able to participate in HR and is a key way to repair double‐stranded DNA breaks.^51^ BLM mutations can cause genome instability and increase the risk of cancer.^52^ Many reports have indicated that nonsense mutations in the BLM gene can increase the risk of prostate cancer.^53^ Not only that, but BLM expression also has a certain correlation with ovarian cancer. Recently, studies have shown that increased expression of BLM is associated with platinum in ovarian cancer. Birkbak et al showed that the average expression level of BLM in carboplatin‐sensitive ovarian cancer was significantly higher from an ovarian cancer gene expression data set. And loss of RAD51 expression at the site of DNA damage made BLM overexpressing cells sensitive to cisplatin. Ultimately, the result represented that BLM was one of the potential predictive biomarkers for platinum sensitivity in ovarian cancer.^40^

KDM1A (also known as LSD1) is the first histone demethylase to be discovered.^54^ As an epigenetic regulator, it regulates normal cell differentiation, gene activation, tumorigenesis and progression.^55^ Several articles have reported that the biological behaviour of KDM1A has a strong correlation with various types of cancers.^56^ Studies on ovarian cancer have indicated that KDM1A is overexpression in tumour tissue. It is associated with FIGO stage or lymphatic metastasis, and patients with lower expression have a shorter overall survival time.^57^, ^58^ Furthermore, KDM1A plays an important role in proliferation, invasion and metastasis in ovarian cancer.^56^, ^59^, ^60^ Some studies have suggested that inhibiting the chemical activity of KDM1A may be a candidate method for cancer treatment.^57^ For example, the combination of trichostatin A and decitabine inhibits KDM1A expression and weakens the migration and invasion of ovarian cancer cells.^61^ Previous reports have pointed out that the reduced expression of KDM1A could prompt autophagy activation.^62^ S2101, one of the most effective KDM1A inhibitors, can suppress ovarian cancer cell viability and stimulate apoptosis and autophagy by regulating the expression level of signalling pathways.^63^ Therefore, according to our research and previous evidence, KDM1A may be a novel candidate signature for the mechanism of platinum response in ovarian cancer.

POLD1 belongs to the family of human DNA polymerases, with a 3′‐5′ exonuclease activity.^64^ Some studies publicly have proved that POLD1 is involved in the regulation of cell proliferation and cell cycle.^65^ And it plays a central role in chromosomal DNA replication, repair and recombination.^66^ Through the molecular phenotype of DNA hypermutation, germline or somatic mutation of POLD1 can cause DNA repair defects and carcinogenesis.^67^ Previous reports have pointed out that POLD1 genetic mutations are inseparable from colorectal cancer,^68^ endometrial cancer^69^ and epithelial cancer.^70^ A study of platinum‐based chemotherapy in non‐small cell lung carcinoma showed that continuous signals were observed in POLD1 in the nucleotide excision repair pathway. SNPs in POLD1 were significantly associated with overall survival and neutropenia.^71^ It was proposed that POLD1 was directly or indirectly related to platinum resistance in mesothelioma.^72^ It has been reported that POLD1 is expressed in a variety of tumour cells, such as breast cancer,^73^ hepatocellular carcinoma,^74^ head and neck cell cancer^75^ and cervical cancer.^76^ As for oesophageal squamous cell carcinoma, POLD1 mediated the chemical resistance to cisplatin through the regulation of HSP90/ERK signalling.^77^

We employed equal weight and random walk algorithm in complex disease‐specific networks so that the analysis results were not biased. The six candidate genes were verified in the multiple validation sets, making these results more accurate. Further exploration of six candidate genes revealed that the mRNA and protein expression levels possessed significant differences in the analysis of clinicopathological factors. Notably, there were also meaningful relationships between these genes and both overall survival and disease‐free survival. The RT‐qPCR assay further confirmed that the expression levels of the six candidate genes in platinum‐sensitive ovarian cancer cell line were higher than those in platinum‐resistant cell line. Therefore, these reliable results show that our analytical method is of great significance in identifying platinum response biomarkers for ovarian cancer.

However, our research still exists some limitations and disadvantages. The RT‐qPCR assay is a sensitive, accurate test method, which can detect the gene expression (ie mRNA) level and perform quantitative analysis.^78^ The Western blotting is the most widely used experimental technique in protein expression and analysis. Strong specificity, high sensitivity and easy operation are its advantages.^79^ In our research, we detected the mRNA expression level and did not verify the protein expression level of the six candidate gene in cisplatin‐resistant and cisplatin‐sensitive ovarian cancer cell lines. The two types of functional genes were used as seed nodes, which have certain limitations. In subsequent studies, we will increase the variety of functional genes as seed nodes and examine the protein expression level of six candidate genes in cell lines. The tissues of ovarian cancer patients with platinum response information will be collected as the research samples. We will strive to provide more valuable research on the mechanism of platinum response in ovarian cancer.

## 5 CONCLUSION

We applied the random walk algorithm based on reconstructed integrated network and analysed the global impact of genes from HR and FA functions. Besides, GSEA enrichment analysis was performed to evaluate the function of the three types of functional genes. The candidate genes were identified and further verified in the three data sets from the GEO database. Moreover, we also performed differentially expressed analysis, clinicopathological multivariate analysis, functional evaluation of mutation neighbouring genes and survival analysis for six candidate genes. Finally, the RT‐qPCR assay was performed to further support the above findings. In conclusion, our research can provide new understandings of the mechanism of platinum response in ovarian cancer patients and identify candidate genes for clinical usage.

## AUTHORS CONTRIBUTIONS

**Ge Lou:** Conceptualization (lead); Funding acquisition (lead); Project administration (lead); Supervision (lead). **Chunlong Zhang:** Conceptualization (lead); Data curation (lead); Formal analysis (lead); Funding acquisition (lead); Project administration (lead); Resources (lead); Supervision (lead); Writing‐original draft (equal); Writing‐review & editing (lead). **Linan Xing:** Conceptualization (lead); Data curation (lead); Formal analysis (lead); Resources (lead); Software (lead); Validation (lead); Writing‐original draft (lead); Writing‐review & editing (lead). **Wanqi Mi:** Data curation (supporting); Writing‐review & editing (supporting). **Yongjian Zhang:** Data curation (supporting); Formal analysis (supporting). **Songyu Tian:** Data curation (supporting). **Yunyan Zhang:** Supervision (supporting). **Rui Qi:** Writing‐review & editing (supporting).

## ETHICS APPROVAL AND CONSENT TO PARTICIPATE

Not applicable.

## CONSENT FOR PUBLICATION

All authors agree for publication.

## COMPETING INTERESTS

The authors declare that they have no competing interests.

## Supporting information

Fig S1

Fig S2

Fig S3

Table S1

Table S2

Table S3

Table S4

## Data Availability

The data used and analysed during this study are available from the corresponding author on request.
