# Supplementary figures and images for: The identification of six risk genes for ovarian cancer platinum response based on global network algorithm and verification analysis
Source: J Cell Mol Med. 2020 Aug 6;24(17):9839–52. doi: 10.1111/jcmm.15567 (PMC7520306; doi:10.1111/jcmm.15567)

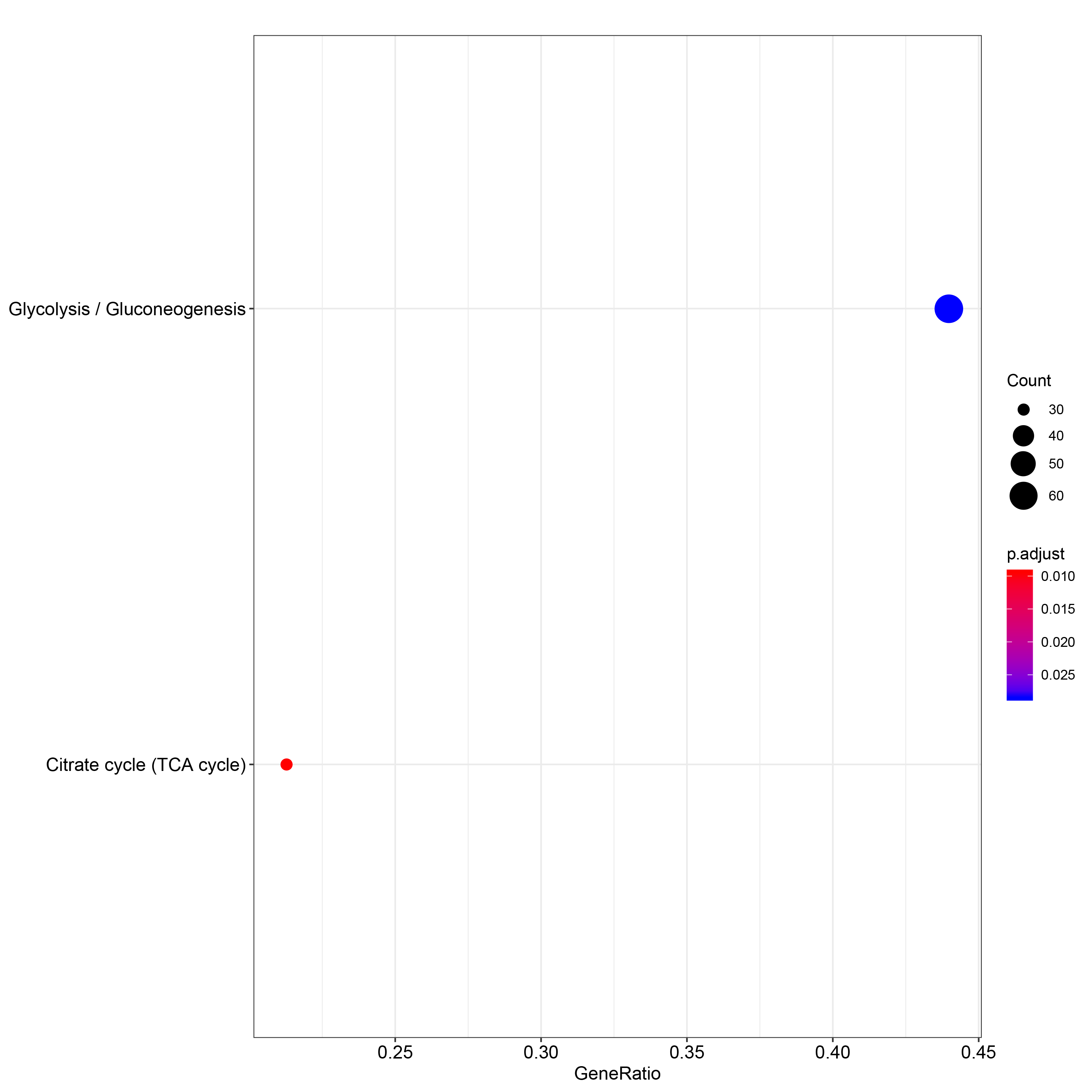

Supplement: Supplementary file 1 — Fig S1 [file JCMM-24-9839-s001.tif]

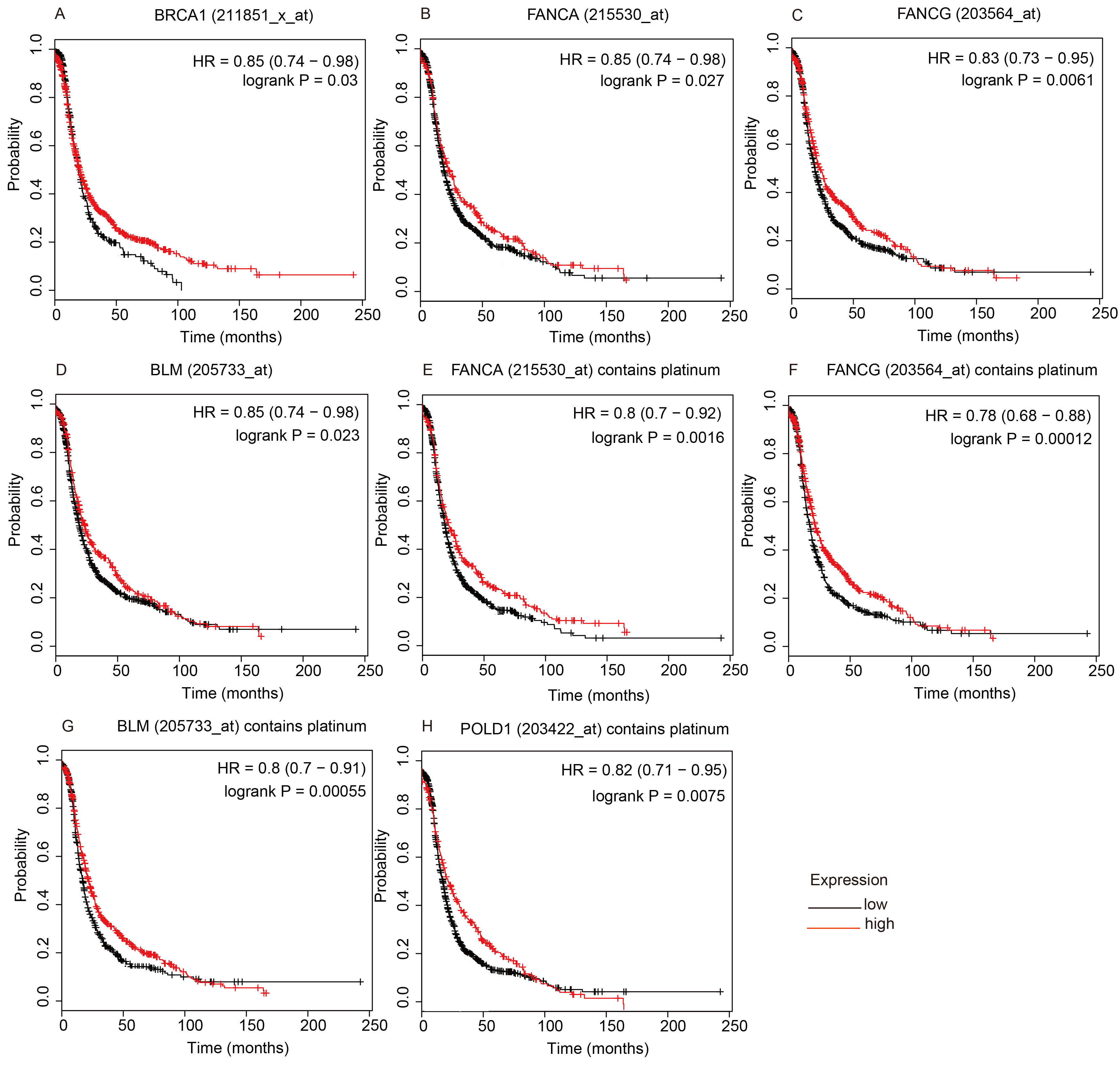

Supplement: Supplementary file 2 — Fig S2 [file JCMM-24-9839-s002.tif]

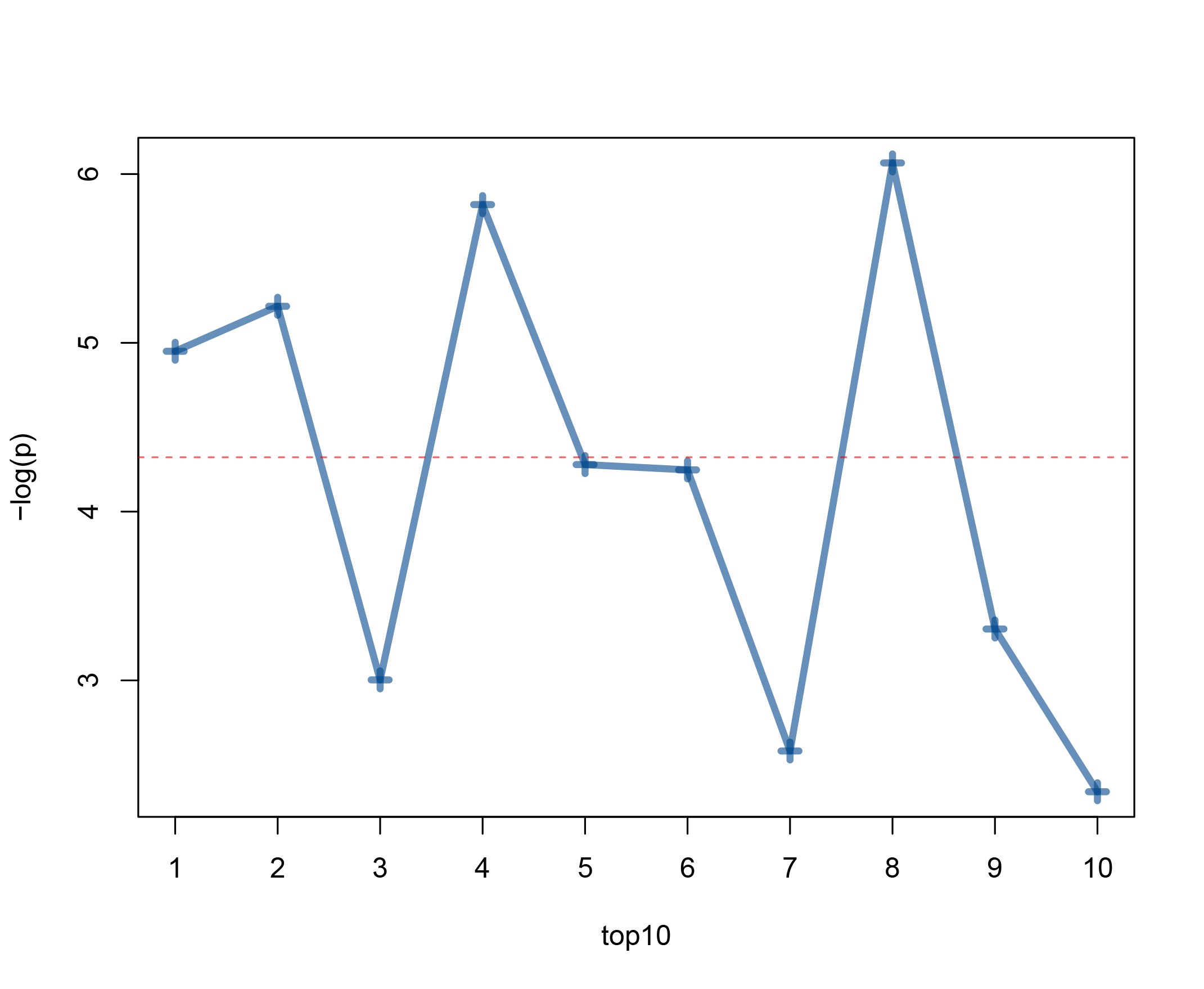

Supplement: Supplementary file 3 — Fig S3 [file JCMM-24-9839-s003.tif]
