# Supplementary material for: The identification of six risk genes for ovarian cancer platinum response based on global network algorithm and verification analysis
Source: J Cell Mol Med. 2020 Aug 6;24(17):9839–52. doi: 10.1111/jcmm.15567 (PMC7520306; doi:10.1111/jcmm.15567)
Supplement: Supplementary file 4 — Table S1 [file JCMM-24-9839-s004.doc]

**The sequences of primers**

| **Gene** | **Primer sequence** |
| --- | --- |
| BRCA1 | forward-5’-GTACAGCTGTGTGGTGCTTCTGT-3’ |
|  | reverse-5’-TCTGCCCAATTGCATGGAAG-3’ |
| FANCA | forward-5’-TGCGAGAGAGAGGAGCTATTGG-3’ |
|  | reverse-5’-CACAAACGTGGAAAGCCTTTG-3’ |
| FANCG | forward-5’-GCCAGGATACCAAAGCCTTACA-3 |
|  | reverse-5’-CCTCCGATCTAGCCTCTTCAGA-3’ |
| BLM | forward-5’-AAGTTTCCTTCTGTTCCGGTGAT-3’ |
|  | reverse-5’-ACACCTGAGGTCTGAGAATCTTCA-3’ |
| KDM1A | forward-5’-ACTCCTGGCCCCTCGATTC-3’ |
|  | reverse-5’-CTCAGCAGAGCACCATGCA-3’ |
| POLD1 | forward-5’-CAGAACTTCGACCTTCCGTACCT-3’ |
|  | reverse-5’-GAAGAGTCCCGGATGTTGGA-3’ |
| GAPDH | forward-5’-CACCCACTCCTCCACCTTTGA-3’ |
|  | reverse-5’-ACCACCCTGTTGCTGTAGCCA-3’ |
